# Evaluating eravacycline’s efficacy against pediatric carbapenem-resistant *Klebsiella pneumoniae* in China: a 6-year study

**DOI:** 10.1128/spectrum.02482-25

**Published:** 2025-11-21

**Authors:** Mingming Zhou, Xiucai Zhang, Shixing Liu, Jintao Xia, Chao Fang, Yining Zhao, Hongqiang Shen

**Affiliations:** 1 Department of Clinical Laboratory, Children’s Hospital, Zhejiang University School of Medicine, National Clinical Research Center for Child Health, National Children’s Regional Medical Center, Hangzhou, Zhejiang, China; Universita degli Studi dell'Insubria, Varese, Italy

**Keywords:** eravacycline, tigecycline, carbapenem-resistant *Klebsiella pneumoniae*, CRKP, children

## Abstract

Eravacycline (ERV) has demonstrated promising *in vitro* antibacterial activity against carbapenem-resistant *Klebsiella pneumoniae* (CRKP). Nonetheless, its clinical application in pediatric cases remains underexplored. Therefore, we assessed the *in vitro* activities of ERV and tigecycline (TGC) against CRKP isolates obtained from pediatric patients in China between 2019 and 2024. The susceptibility rates of the 74 CRKP strains to TGC and ERV were 100% and 87.8%, respectively. Both TGC and ERV had MIC ranges of 0.125–2 μg/mL, with MIC_50_ at 0.25 µg/mL and MIC_90_ at 1 µg/mL. Statistically significant differences in ERV susceptibility were observed across different carbapenemases, sequence types (STs), and age groups, with the type of carbapenemase emerging as the primary determinant. The lowest susceptibility was noted for *bla_KPC-2_* (64.3%) and ST11 (54.6%), while *bla_NDM-1_* (97.4%), ST25, and ST17 (100%) exhibited the highest. The lowest susceptibility was observed in CRKP strains isolated from >3-year group (46.7%), whereas strains from both neonates and toddlers exhibited 100% susceptibility. When the MIC was below 0.5 µg/mL, ERV exhibited higher inhibition rates against CRKP compared to TGC. However, at MIC values of 0.5, 0.75, 1.5, and 2 µg/mL, the inhibition rates of both drugs were comparable. Among CRKP strains carrying *bla_KPC-2_*, *bla_NDM-1_*, and *bla_NDM-5_*, both the MIC_50_ and MIC_90_ values of ERV were less than or equal to TGC’s. ERV consistently had lower or equal MIC_90_ values compared to TGC for common STs, except for ST11, where ERV’s MIC_50_ was also lower or equal. ERV demonstrated significant *in vitro* activity against pediatric CRKP isolates collected over a 6-year period, supporting its potential benefits in treating pediatric CRKP infections, although further *in vivo* validation is necessary.

**IMPORTANCE:**

Eravacycline (ERV) has demonstrated promising *in vitro* antibacterial activity against carbapenem-resistant *Klebsiella pneumoniae* (CRKP). Current research predominantly addresses adult populations, leaving its clinical application in pediatric cases insufficiently explored. Given that the resistance mechanisms of CRKP in pediatric patients differ from those in adults, primarily due to the production of metallo-β-lactamases, and considering that ERV, like tigecycline, may be used off-label in pediatric populations, this study was conducted. The results demonstrate that ERV exhibited significant *in vitro* efficacy against pediatric CRKP isolates. However, unlike previous studies, the susceptibility rates of ERV vary considerably across different carbapenemases, sequence types, and age groups of CRKP, with the type of carbapenemase emerging as the primary determinant. These findings suggest that ERV has promising *in vitro* activity against CRKP isolates from pediatric patients, indicating potential clinical applicability for this demographic.

## INTRODUCTION

Carbapenem-resistant *Klebsiella pneumoniae* (CRKP) is increasingly acknowledged as a significant public health threat. In the 2024 World Health Organization bacterial priority pathogens list, CRKP received the highest overall ranking (1). CRKP is capable of causing a range of severe diseases in pediatric populations, including bloodstream infections, pneumonia, meningitis, and urinary tract infections (2). Once children are infected with CRKP, the mortality rate is elevated, and the prognosis is poor (3). The rise in CRKP infections among children is particularly concerning due to the limited availability of effective antibiotics and the negative impact on clinical outcomes.

Tigecycline (TGC), a glycylcycline antibiotic, has been employed off-label in these cases, despite its initial approval for the treatment of complicated skin and intra-abdominal infections in adults. The off-label application of TGC in pediatric patients is motivated by its broad-spectrum efficacy against multidrug-resistant organisms, including CRKP, which exhibit resistance to numerous other antibiotics (4). In addition to TGC, antibiotics such as colistin and polymyxin B are considered last-resort options for the treatment of CRKP infections. The administration of these antibiotics is associated with the risk of resistance development, thereby complicating treatment strategies. The combination of TGC with other antibiotics, such as colistin, has been explored to mitigate resistance development and enhance treatment outcomes (5). Ceftazidime-avibactam (CZA) has shown efficacy in treating CRKP infections. However, it is ineffective against metallo-β-lactamase (MBL)-producing strains, limiting its applicability in certain clinical scenarios. This limitation is particularly pronounced in pediatric patients, where the prevalence of MBL-positive CRKP strains is relatively high, further constraining the use of CZA (6). Additionally, CRKP has been observed to develop co-resistance to other antibiotics, such as colistin and cefiderocol, under selective pressure (7). This co-resistance further restricts treatment options and highlights the necessity for novel therapeutic strategies and comprehensive surveillance systems to monitor and control the spread of CRKP.

Eravacycline (ERV), a next-generation tetracycline derivative, has emerged as a promising solution due to its unique chemical structure, which enhances its potency and broadens its spectrum of activity. ERV has exhibited significant antibacterial activity against carbapenem-resistant gram-negative bacteria *in vitro*, including CRKP (8). Current research predominantly targets adult populations (9–12), leaving the clinical application of these findings in pediatric cases insufficiently explored. Given the substantial physiological differences between children and adults, as well as differences in CRKP resistance mechanisms (13), further investigation is essential to ascertain the safety and efficacy of these findings for pediatric clinical applications.

The off-label use of antibiotics in pediatric populations is not uncommon, as evidenced by studies showing high rates of off-label and unlicensed drug use in pediatric and neonatal intensive care units. These practices are often necessitated by the lack of pediatric-specific clinical trials and approved medications (14). Although ERV has not yet received approval for pediatric indications, real-world studies have demonstrated its adaptability and effectiveness across various clinical scenarios, underscoring its potential utility beyond approved indications (15). Therefore, conducting comprehensive research of ERV focusing on pediatric populations will help bridge the gap between *in vitro* efficacy and clinical application, ensuring that children receive effective and safe treatment options for these challenging infections.

## RESULTS

In this study, a total of 74 CRKP strains, collected between 2019 and 2024, were analyzed. The majority of isolates were obtained from infant patients aged 29 days to 1 year (47/74, 63.5%), with 51.4% (38/74) of the patients being male. The primary source of the isolates was respiratory specimens, including bronchoalveolar lavage fluid and sputum (49/74, 66.2%), followed by urine (10/74, 13.5%) and pus (9/74, 12.2%). Carbapenemase-encoding genes were identified in 64 of the 74 CRKP isolates. Among these, *bla_NDM-1_* was the most prevalent gene (38/64, 59.4%), followed by *bla_KPC-2_* (14/64, 21.9%), *bla_NDM-5_* (11/64, 17.2%), *bla_IMP-4_* (1/64, 1.6%), and *bla_OXA-48_* (1/64, 1.6%). Notably, one CRKP strain harbored both *bla_NDM-5_* and *bla_IMP-4_*. A total of 22 sequence types (STs) were identified among the 74 CRKP strains, with the predominant STs being ST25 (29/74, 39.2%), ST11 (11/74, 14.9%), ST15 (5/74, 6.8%), ST17 (5/74, 6.8%), and ST348 (5/74, 6.8%; Table 1). Additionally, a novel ST (ST8234) was detected.

**TABLE 1:** Susceptibility results of ERV and TGC in CRKP isolated from children

Characteristics (*n*)	ERV (μg/mL)					TGC (μg/mL)			
	MIC_50_	MIC_90_	MIC range	Susceptibility (%)	*P*	MIC_50_	MIC_90_	MIC range	Susceptibility (%)
Carbapenemase gene^*a*^									
*bla_KPC-2_* (14)	0.19	1	0.125–2	64.3	0.004	0.25	1	0.125–1.5	100
*bla_NDM-1_* (38)	0.25	0.38	0.125–1	97.4		0.25	0.5	0.125–1.5	100
*bla_NDM-5_* (11)	0.38	0.5	0.125–1.5	90.9		0.38	1	0.25–2	100
STs^*b*^									
ST25 (29)	0.25	0.38	0.125–0.5	100.0	0.002	0.25	0.5	0.125–1.5	100
ST11 (11)	0.38	1	0.125–2	54.6		0.25	1	0.125–1.5	100
ST15 (5)	0.25	1.5	0.125–1.5	80.0		0.38	1.5	0.25–1.5	100
ST17 (5)	0.19	0.38	0.125–0.38	100.0		0.25	0.38	0.19–0.38	100
ST348 (5)	0.38	1.5	0.25–1.5	80.0		0.38	2	0.38–2	100
Ages									
≤28 days (9)	0.38	0.38	0.19–0.38	100.0	0.000	0.25	0.5	0.25–0.5	100
29 days to 1year (47)	0.25	0.5	0.125–1.5	97.9		0.25	0.5	0.125–2	100
>1–3 years (3)	0.38	0.5	0.38–0.5	100.0		0.5	0.5	0.38–0.5	100
>3 years (15)	0.75	1.5	0.125–2	46.7		0.5	1.5	0.19–1.5	100
All isolates (74)	0.25	1	0.125–2	87.8		0.25	1	0.125–2	100

^*a*^ Only analyze the predominant carbapenemase genes.

^*b*^ Only analyze the predominant STs.

**Fig 1:**
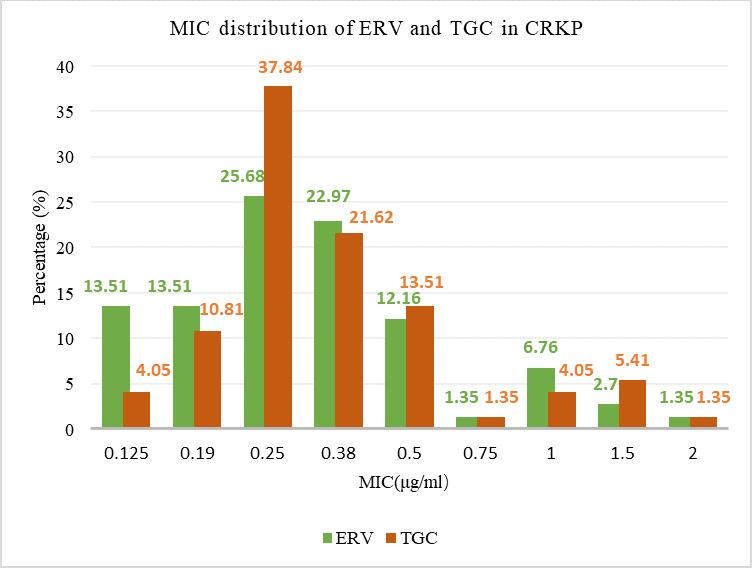
MIC distribution of ERV and TGC in CRKP isolated from children.

**Fig 2:**
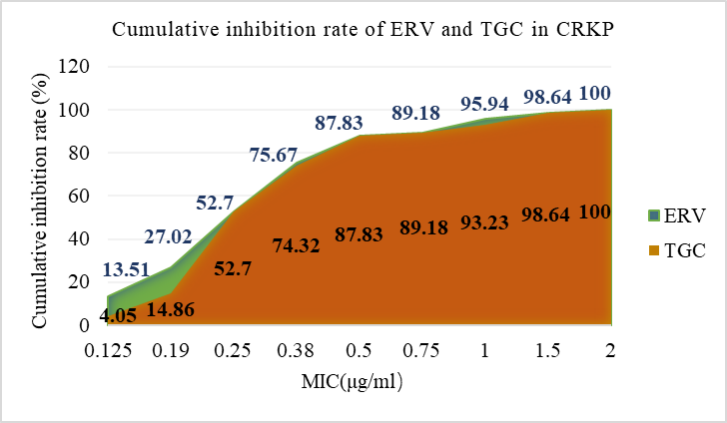
Cumulative inhibition rate of ERV and TGC in CRKP isolated from children.

**Fig 3:**
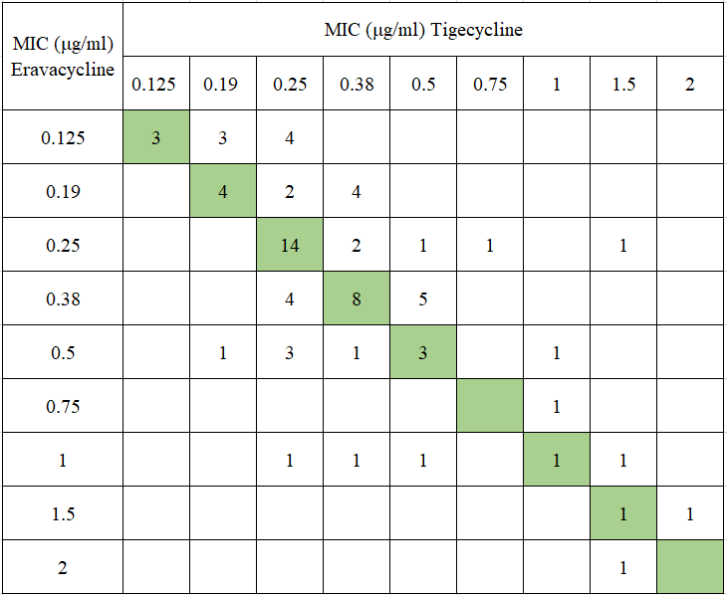
Interrelationship between MICs of ERV and TGC for the CRKP isolates from children. Green boxes represent the line of equivalence. Numbers above this line indicate ERV is more active in these isolates, while numbers below indicate TGC is more active.

The susceptibility of 74 CRKP strains to TGC and ERV was 100% and 87.8%, respectively. The MIC range for both TGC and ERV was 0.125–2 μg/mL, with MIC_50_ and MIC_90_ values of 0.25 µg/mL and 1 µg/mL, respectively, for each (Table 1). The MIC values for ERV were predominantly distributed at 0.25 µg/mL and 0.38 µg/mL, comprising approximately 50% of the total. TGC demonstrated a similar distribution pattern but with a higher proportion concentrated at 0.25 µg/mL (Fig. 1). When the MIC was below 0.5 µg/mL, ERV inhibited more CRKP strains than TGC. However, at MIC values of 0.5 µg/mL, 0.75 µg/mL, 1.5 µg/mL, or 2 µg/mL, both antibiotics exhibited equivalent inhibition rates against CRKP (Fig. 2). There were 34 strains with identical MICs for ERV and TGC, representing 45.9% of the total. The number of strains with lower MICs for ERV compared to TGC was 27, accounting for 36.5%, whereas 13 strains exhibited higher MICs for ERV than for TGC, accounting for 17.6% (Fig. 3).

For CRKP strains harboring *bla_KPC-2_*, *bla_NDM-1_*, and *bla_NDM-5_*, the susceptibility to TGC remained consistently at 100%, while ERV susceptibility varied significantly, with *bla_KPC-2_* showing the lowest susceptibility (64.3%) and *bla_NDM-1_* the highest (97.4%). In CRKP strains harboring *bla_KPC-2_, bla_NDM-1_,* and *bla_NDM-5_*, both the MIC_50_ and MIC_90_ values for ERV were equal to or lower than those for TGC. Notably, CRKP strains with *bla_KPC-2_* demonstrated the lowest MIC_50_ but the highest MIC_90_ for both ERV and TGC (Table 1).

Among CRKP of various STs, TGC exhibited 100% susceptibility, whereas ERV susceptibility varied significantly, with the lowest rate in ST11 (54.6%) and the highest in ST25 and ST17 (100%). In common STs of CRKP, the MIC_90_ values for ERV were consistently equal to or lower than those for TGC. Except for ST11, the MIC_50_ values for ERV in other STs were also equal to or lower than those for TGC (Table 1).

TGC maintained 100% susceptibility across CRKP isolates from all age groups, whereas ERV susceptibility showed significant variation. The lowest susceptibility was observed in CRKP strains isolated from >3-year group (46.7%), whereas strains from both neonates and toddlers exhibited 100% susceptibility. Notably, CRKP isolates from the >3-year group exhibited the highest MIC_50_ and MIC_90_ values for both ERV and TGC, whereas the infant group showed the lowest MIC_50_ and MIC_90_ values (Table 1).

## DISCUSSION

This study investigated CRKP strains harboring a variety of carbapenemases in pediatric patients, in contrast to adult cases, which predominantly harbor KPC; the focus was on MBLs. The study assessed the efficacy of ERV across CRKP isolates with differing carbapenemase types, STs, and age groups and conducted a comparative analysis with TGC. The findings revealed promising *in vitro* activity of ERV against CRKP isolates from pediatric patients, suggesting potential clinical applicability for this demographic.

The susceptibility rate of 74 CRKP strains to ERV was 87.8%, surpassing the adult susceptibility rate in the same region (53.1%) (8) and in other regions of China (84%) (16). The MIC range for ERV was 0.125–2 μg/mL, which was lower than that observed in adults in the same region (≤0.06–16 μg/mL) (8) and in the USA (0.125–8 μg/mL) (17). Previous studies have indicated that the MIC_50_ of CRKP strains ranges from ≤0.5 to 1 µg/mL, while the MIC_90_ ranges from 1 to 8 µg/mL (15). In contrast, the MIC_50_ (0.25 µg/mL) and MIC_90_ (1 µg/mL) of ERV in CRKP isolates from children in this study were relatively low. These results consistently demonstrate that ERV exhibits enhanced efficacy in pediatric populations. In studies examining carbapenem-non-susceptible Enterobacterales, the MIC_50_ of ERV was consistently reported to be ≤0.5 µg/mL, regardless of the specific carbapenemase present (15), a finding that aligns with the results of this study. Previous research has demonstrated that the bactericidal efficacy of ERV is not significantly affected by the type of carbapenemase, whether KPC-carbapenemase, MBL, or co-producers, under *in vitro* conditions (16). In that study, the susceptibility rates of ERV were 84% for KPC-2 producers and 77% for MBL producers. However, in our study, the susceptibility of ERV varied among different carbapenemases, with *bla_KPC-2_* exhibiting the lowest susceptibility at 64.3%, whereas *bla_NDM_* exceeded 90%. These discrepancies may be attributed to differences in study populations and the distribution of carbapenemase types. In the previous study, CRKP strains carrying *bla_KPC-2_* were predominant (156/183, 85.2%), while *bla_NDM_* were less prevalent (17/183, 9.3%) (16). In contrast, the current study found a predominance of *bla_NDM_* (78.2%), although *bla_KPC-2_* still represented a significant proportion (21.9%). In this study, the susceptibility rate of CRKP strains harboring *bla_NDM_* to ERV was found to be higher in pediatric populations compared to adults in Turkey (66.7%, MIC_50_: 0.5 µg/mL, MIC_90_: 2 µg/mL) (18) and in Greece (61.3%, MIC_50_: 0.5 µg/mL, MIC_90_: 1 µg/mL) (19). ERV demonstrated variable sensitivity rates across different STs, with ST25 and ST17 exhibiting the highest rates (both 100%), whereas ST11 showed the lowest rate (54.6%). Further analysis indicated that ST25 isolates exclusively carried *bla_NDM-1_*, ST17 harbored both *bla_NDM-5_* and *bla_NDM-1_*, and ST11 were entirely *bla_KPC-2_* positive, which aligns with the susceptibility patterns associated with different carbapenemases. This suggests that the observed differences among STs are predominantly attributable to the specific types of carbapenemases present. Notably, ST11-CRKP is recognized for its high virulence (20), highlighting the need to consider the reduced sensitivity of ERV when managing such infections in pediatric patients. Moreover, ERV susceptibility varies across age groups, with the lowest rate observed in those >3 years (46.7%). Among these cases, 53.3% were attributed to KPC-2. This indicates that the differences between age groups are primarily due to the types of carbapenemases present.

In the present study, ERV exhibits a lower overall susceptibility rate compared to TGC (87.8% vs. 100%); however, the MIC range, MIC_50_, and MIC_90_ values remain consistent between the two agents. Under identical MIC conditions, ERV exhibited higher inhibition rates against CRKP relative to TGC, corroborating previous research findings (21). In this study, 36.5% of CRKP strains had a lower MIC for ERV compared to TGC, while 45.9% of strains showed equal MIC values for both antibiotics. These results suggest that, under equivalent MIC conditions, ERV possesses stronger *in vitro* antibacterial activity than TGC against CRKP in pediatric cases.

Prior studies have indicated that ERV’s potency is twofold to fourfold greater than that of TGC against Enterobacterales (9, 10, 22). Additionally, other research has demonstrated that ERV exhibits stronger bactericidal effects against carbapenem-resistant gram-negative bacteria harboring *bla_KPC-2_*, *bla_OXA-23_*, *bla_NDM-1_*, and *bla_NDM-16_* compared to TGC (8). Consistently, among CRKP strains carrying *bla_KPC-2_, bla_NDM-1_,* and *bla_NDM-5_* in this study, both the MIC_50_ and MIC_90_ of ERV were less than or equal to those of TGC. Specifically, for CRKP isolates carrying *bla_KPC-2_*, the MIC_50_ and MIC_90_ of ERV in this study were lower than those in adults from the same region (0.125 µg/mL and 1 µg/mL vs 0.5 µg/mL and 2 µg/mL). Similarly, the MIC_50_ and MIC_90_ values for TGC in pediatric populations were lower than those observed in adults (0.25 µg/mL and 1 µg/mL vs 1 µg/mL and 2 µg/mL) (8), suggesting that both TGC and ERV may offer enhanced efficacy in treating CRKP infections harboring *bla_KPC-2_* in children.

Several limitations of the current study warrant consideration. Primarily, the investigation is confined to the *in vitro* activity of ERV in CRKP isolates from children. Given the paucity of data concerning the use of ERV in pediatric populations, the study does not encompass clinical application data, such as clinical outcomes and *in vivo* efficacy in children. Although our research offers *in vitro* experimental evidence supporting the potential clinical application of ERV in pediatric settings, it is important to recognize that ERV has not been approved for use in children. Extending its application to this vulnerable group poses numerous challenges, including concerns about safety, complexities in pediatric dosing, and regulatory issues associated with off-label use. Consequently, comprehensive *in vivo* studies in pediatric cohorts are essential to acquire robust data on safety and efficacy.

### Conclusion

ERV exhibited notable *in vitro* efficacy against pediatric CRKP isolates, achieving an overall susceptibility rate of 87.8%. However, the susceptibility rates of ERV vary significantly across different carbapenemases, STs, and age groups of CRKP, with the type of carbapenemase emerging as the primary determinant. These *in vitro* findings underscore the significant clinical potential of ERV as a treatment for pediatric CRKP infections, although further *in vivo* validation is necessary.

## MATERIALS AND METHODS

### Strain collection and identification

CRKP isolates were collected at Children’s Hospital, Zhejiang University School of Medicine, from January 2019 to December 2024. All CRKP isolates were identified using Matrix-Assisted Laser Desorption Ionization Time of Flight Mass Spectrometry (MS, Bruker, Germany).

### Antimicrobial susceptibility test

The antimicrobial susceptibility testing was conducted utilizing the commercial micro-dilution method (VITEK 2 COMPACT, BioMérieux, France). The susceptibilities of ERV and TGC were assessed using MIC Test Strips (Liofilchem, Italy). The interpretation of susceptibility results adhered to the Clinical Laboratory Standards Institute guidelines M100 for all agents except ERV and TGC, for which the U.S. Food and Drug Administration breakpoints were applied. Quality control was ensured using *Escherichia coli* ATCC25922 and *Pseudomonas aeruginosa* ATCC27853.

### Whole genome sequencing

Genomic DNA was extracted with the QIAGEN DNA miniprep kit (QIAGEN, Germany), and the extracted DNA was subsequently sent to Hangzhou Digital-Micro Biotechnology Co., Ltd. for sequencing. Whole genome sequence was executed on the Illumina Hiseq X Ten platform employing a 2 × 150 bp Paired-End configuration. Sequencing reads were trimmed and assembled *de novo* into contigs using the Unicycler pipeline v0.4.8 (https://github.com/rrwick/Unicycler) (23). Resistance genes were identified using Abricate (https://github.com/tseemann/abricate) with the Resfinder database. Multilocus sequence typing analysis was conducted using the mlst tool (https://github.com/tseemann/mlst).

### Statistical analyses

All statistical analyses were performed using the Statistical Package for the Social Sciences, Version 22.0 (IBM Corp., Armonk, NY, USA). Fisher’s exact test was utilized to compare the susceptibility of ERV across different groups, with a *P*-value of less than 0.05 considered indicative of statistical significance.
